# Preparation of inocula for experimental infection of blood with *Streptococcus pneumoniae*

**DOI:** 10.1016/j.mex.2015.11.003

**Published:** 2015-11-21

**Authors:** Santiago Vivas-Alegre, Isabel Fernández-Natal, Eduardo López-Fidalgo, Octavio Miguel Rivero-Lezcano

**Affiliations:** a Servicio de Digestivo, Hospital de León, Altos de Nava s/n, 24008 León, Spain; b Institute of Biomedicine (IBIOMED), University of León, León, Spain; c Servicio de Microbiología, Hospital de León, Altos de Nava s/n, 24008 León, Spain; d Unidad de Investigación, Hospital de León, Altos de Nava s/n, 24008 León, Spain; e Fundación Instituto de Estudios de Ciencias de la Salud de Castilla y León, Spain

**Keywords:** Autolysis, Cryoprotectant, *Escherichia coli*, Plasma, Virulence, Whole blood

## Abstract

Experimental infections of either cells or animals require the preparation of good quality inocula. Unfortunately, the important pulmonary pathogen *Streptococcus pneumoniae* is a fastidious microorganism that suffers an autolysis process when cultured in vitro. Supplementation of Todd–Hewitt broth with a biological buffer (20 mM Tris–HCl, pH = 7.8) promotes a six hours delay in the beginning of the autolysis process. Additional improvements include washing bacteria before freezing, avoiding manipulations after thawing, and the use of glycerol (<18%) as a cryoprotectant, instead of reagents like skimmed milk that may affect cell cultures. With the proposed protocol >70% of the frozen bacteria was viable after 28 weeks at −80 °C, and aliquots were highly homogeneous. We have tested their utility in a whole blood infection model and have found that human plasma exhibits a higher microbicidal activity than whole blood, a result that we have not found previously reported. Additionally, we have also observed significant variations in the antimicrobial activity against different strains, which might be related to their virulence.

• Media culture buffering extends *S. pneumoniae* viability for 6 h.
• Washing before freezing of single use aliquots minimizes manipulation after thawing.
• Experimental infection with the frozen inocula has shown that plasma has higher bactericidal activity than blood.

## Graphical abstract

**Figure:**
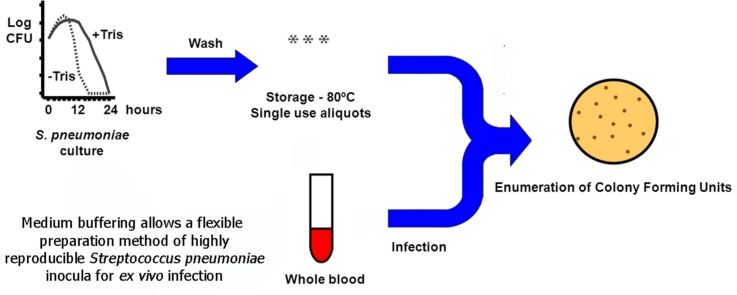

## Method details

1. Seed the whole surface of a blood agar plate with *Streptococcus pneumoniae* from stock and incubate overnight at 37 °C. Other media that allow the growth of the bacterium, like Todd–Hewitt agar plates are also appropriate.
2. Harvest the bacteria and inoculate 40 ml of buffered Todd–Hewitt broth. Composition: Todd–Hewitt broth supplemented with 2% yeast extract and 20 mM Tris(hydroxymethylaminoethane (Tris, p*K*_a_ = 8.1). Adjust pH with HCl to 7.8 before sterilization.
3. Incubate the culture for 6–10 h at 37 °C in an incubator at 200 r.p.m. The viability of the bacteria remains high for this period of time in buffered Todd–Hewitt broth.
4. Centrifuge culture at 10,000 × *g* at 6 °C for 5 min in sterile conditions and discard supernatant. From this point, bacteria should always be below 6 °C, until they are frozen at −80 °C.
5. Wash bacteria twice with cold phosphate buffered saline (PBS) by centrifuging at 10,000 × *g* at 6 °C for 5 min. Bacterial wash after freezing of the stock results in a higher variability in the number of recovered viable bacteria.
6. Suspend bacteria in 1200 μl of cold RPMI-1640/15% glycerol.
7. Freeze single use aliquots at −80 °C. The stock concentration usually is above 10^9^ bacteria/ml. Quantify the colony forming units (CFU) as described below one week after freezing because the bacterial death rate is high in the first days.

### Experimental infection of whole blood

A modification of the direct bactericidal test of Lancefield [1] was performed.

8. Collect blood from healthy donors, following informed consent and approval of the protocol by the institution Clinical Research Ethics Board. Clotting was inhibited by K_3_-ethylenediaminetetraacetic acid (EDTA). We have not tested other inhibitors.
9. Dilute biological samples (blood, plasma or serum, 40%) with RPMI-1640 medium (60%) and place 200 μl in 2 ml microtubes.
10. Infect diluted samples with 10^3^ bacteria/100 μl and incubate at 37 °C in a rotator (20 r.p.m.) for the indicated times. Slightly different rotator speeds do not influence the outcome of the experiment.
11. After incubation, dilute 10 μl of infected blood in 90 μl of water and lyze cells in an ultrasonic bath for 3 min. We use a 35 kHz ultrasonic bath (half wave mode).
12. Inoculate 50 μl of the released bacteria in 55 mm diameter agar plates (Todd–Hewitt broth with 0.5% yeast extract and 1.5% agar). After absorption, add an agar overlay (Todd–Hewitt broth containing 0.5% yeast extract/0.75% agar) supplemented with 100 mg/ml 2,3,5,-triphenyltetrazolium chloride, a dye indicative of cellular metabolism that stains the colonies deep red [2]. Enumerate CFU after an overnight incubation. *S. pneumoniae* colonies are small and difficult to visualize. The indicative dye facilitates the detection and enumeration of colonies.

### Development of the method

**Fig. 1:**
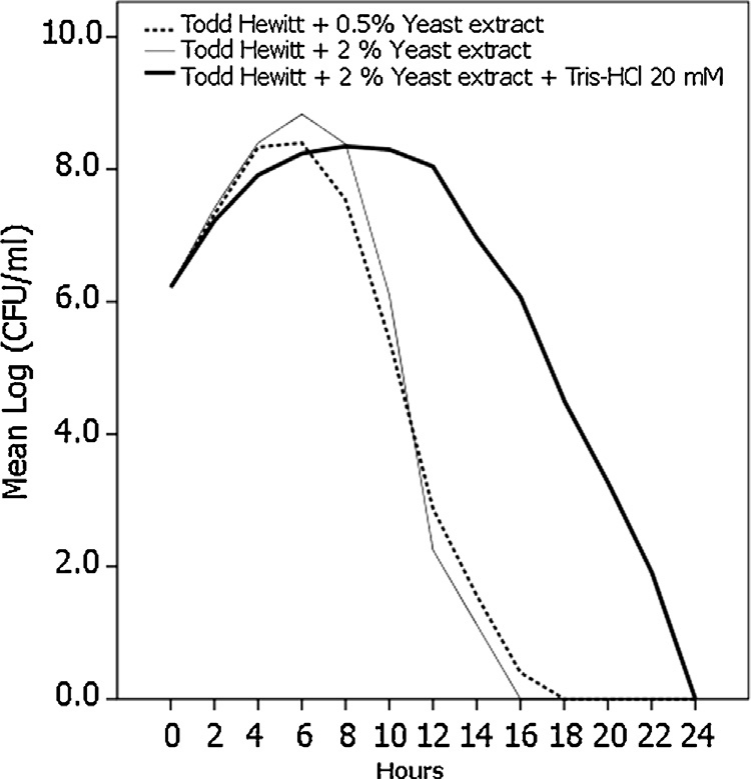
Influence of yeast extract and Tris–HCl supplementation in *S. pneumoniae* growth. Todd–Hewitt broth supplemented with either yeast extract (0.5 or 2%) or 20 mM Tris–HCl, pH = 7.8 (2 ml) was seeded with 10^6^ bacteria. Every two hours 10 μl were decimally diluted and plated in Todd–Hewith agar and overlaid with Todd–Hewith agar with TTC. Data represent the mean log CFU from four independent experiments.

Medium acidification plays an important role in *S. pneumoniae* autolysis and therefore we tested whether the addition of biological buffers at pH = 7.8 would diminish or delay the process. We tried three buffers: 3-(N-morpholino)propanesulfonic acid (MOPS, p*K*_a_ = 7.20); 2(4-(2-hydroxyethyl)-1-piperazine)-ethanesulfonic acid (HEPES, p*K*_a_ = 7,55) and Tris(hydroxymethylaminoethane (Tris, p*K*_a_ = 8.1), adjusted with NaOH or HCl. While we found that the former buffers were toxic for the bacteria, Tris–HCl allowed *S. pneumoniae* growth. When bacteria were inoculated in Todd–Hewitt broth we observed that the medium supplemented with 2% yeast extract allowed more growth than with 0.5%, but in both cases the beginning of the autolysis process occurred immediately after reaching the maximum growth point, at 6 h for this strain (Fig. 1). Todd–Hewitt broth supplemented with 2% yeast extract and, additionally, with 20 mM Tris–HCl also reached a high level of growth at 6 h after inoculation, but in contrast with broth without Tris–HCl the autolysis process was delayed for 6 more hours. Using this combination of yeast extract and Tris–HCl, the maximum growth level was slightly lower than in Todd Hewitt/2% yeast extract without Tris–HCl, but similar to that in Todd Hewitt/0.5% yeast extract. This change in the growth curve allows a wider operational window with a diminished risk of manipulating bacteria that have already begun the autolysis process. Without Tris–HCl the optimum point in the curve growth before the beginning of autolysis may vary depending on the strain, but the addition of the buffer provides the confidence that for several hours many strains will not have suffered the autolysis process, avoiding the characterization of the curve growth for each strain. In all cases Todd–Hewitt modified media should be used within two weeks from preparation.

There are difficulties to store *S. pneumoniae* maintaining its viability [3] and many cryoprotectants have been used to preserve microorganisms at freezing temperatures [4]. Nevertheless, we had the purpose to avoid protectants that may affect the cells at the time of infection, precluding e.g. dimethylsulfoxide that may activate cells or skimmed milk, frequently contaminated with bacterial products. For this reason we performed a mixture experiment with RPMI-1640, glycerol and foetal calf serum (FCS) with a range of 0–80% for each of them, using the programme Design-Expert 9 (Stat-Ease, Inc., MN, USA). We kept the bacteria at −80 °C for 10 days, inoculated decimal dilutions in the described Todd–Hewitt agar with TTC overlay and enumerated CFU after an overnight incubation. In our results analysis we applied the goal “maximum number of CFU” and found that two optimal mixtures were 55% RPMI-1640/30% FCS/15% glycerol and 85% RPMI-1640/15% glycerol. We preferred the latter because it avoided the use of FCS, a component that added a complex mixture of biological molecules that might have an influence on infected cells. The model also predicted that ≥18% glycerol decreased the number of recovered bacteria. After 28 weeks 73.2% of the inocula was viable (SD = 0.60, *n* = 4). Freezing in the alternative mixture with FCS allowed a slightly higher survival (76.8% SD = 0.55, *n* = 4), but the difference was not statistically significant (Student's *t*-test, *p* = 0.932).

### Experimental infection of whole blood with the *S. pneumoniae* frozen stock

We analyzed the utility of the whole blood model for characterizing the immunological response to *S. pneumoniae* using frozen stocks from three different strains: the strain 12/11240, isolated from a clinical sample at the Servicio de Microbiología from Hospital de León (Spain), that was identified and serotyped (serotype 15B) by standard procedures, and two collection strains purchased from the Spanish Type Culture Collection (CECT, Colección Española de Cultivos Tipo). CECT 782 (serotype 19F) is designated CCUG 1407 (Culture Collection, University of Göteborg, Sweden). CECT 993 (serotype 1) corresponds to ATCC 33400 (American Type Culture Collection). *Escherichia coli* (CECT 568) corresponds to ATCC 13027, and was kindly provided by Dr. Leandro Rodríguez Aparicio. *E. coli* inocula was prepared as indicated for *S. pneumoniae*, but using the medium LB, both broth and agar, for growth and enumeration of colonies.

Blood was collected from healthy donors, following consent and approval of the protocol by the Hospital of León Clinical Research Ethics Board. Tubes for serum collection included a clot activator (BD Vacutainer tubes, red closure, Becton Dickinson), and for both blood and plasma collection, clotting was inhibited by EDTA (lavender closure, Becton Dickinson). For plasma recovery, 1 ml of blood was centrifuged at 14.000 × *g*, 5 min at room temperature. To ensure that the supernatant (plasma) was free of cells the sample was centrifuged again in the same conditions and supernatant was recovered.

**Fig. 2:**
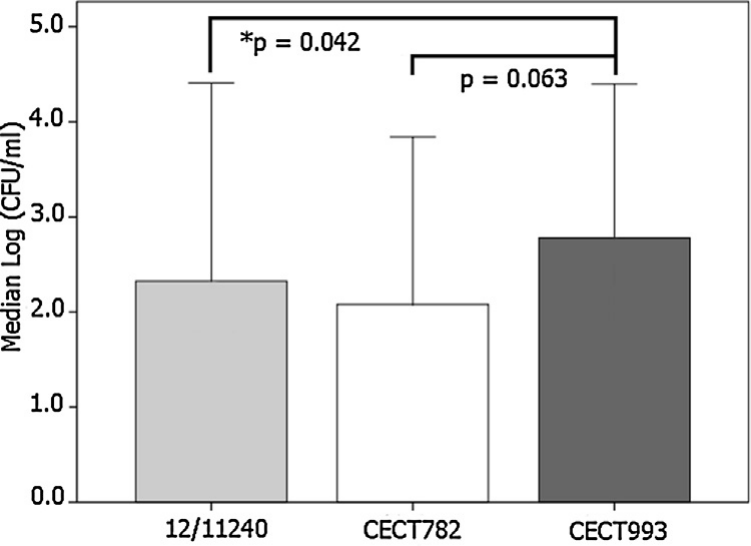
Variability in the antimicrobial activity against different *S. pneumoniae* strains. Whole blood (40%) diluted with RPMI-1640 (60%) was infected with a stock of frozen *S. pneumoniae*. After 240 min, decimal dilutions of samples were plated in Todd–Hewitt agar. Data represent the median log_10_ CFU (95% confidence interval) from 6 independent experiments and was analyzed by the Friedman test. Results from each individual for the 3 strains were grouped in blocks. Pair-wise comparisons with *p < 0.05 were considered significant.

We analyzed whether there were differences in the whole blood antimicrobial activity against unrelated strains. Indeed, CECT 993 remained more viable than the other two strains after 240 min, although it was statistically significant only for 12/11240 (*p* = 0.042, Fig. 2). Consequently, the whole blood model is potentially useful to characterize the virulence of different strains.

**Fig. 3:**
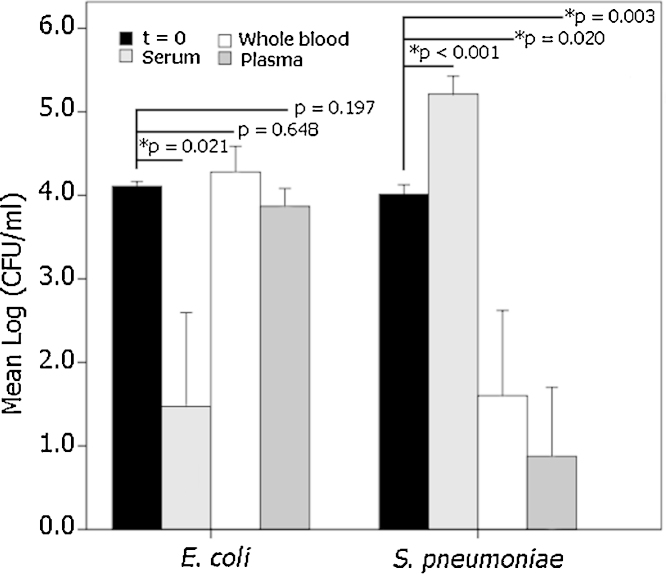
Experimental infection of whole blood, serum and plasma with *S. pneumoniae* CECT 993 and *E. coli*. Samples (whole blood, serum or plasma), were processed as indicated in Fig. 2 for whole blood. Data represent the mean log_10_ CFU ± SD (*n* = 5). Antimicrobial activity was analyzed by ANOVA, and pair-wise comparisons with log_10_ CFU at inoculation (*t* = 0) by the Games–Howell post hoc test was considered significant if *p < 0.05.

We also used the bacterial stocks to characterize the antimicrobial activity of whole blood, serum and plasma against *S. pneumoniae* CECT 993 and *E. coli* (Fig. 3). *S. pneumoniae* grows in serum (*p* < 0.001), but is killed by both whole blood and plasma (*p* = 0.02 and *p* = 0.003, respectively). In fact, plasma exhibits a stronger bactericidal activity than blood (*p* = 0.027, paired Student's *t* test). As a control of the serum antimicrobial activity we also infected the three models with *E. coli*, a Gram-negative microorganism, and the bacterium was successfully killed (*p* = 0.021, ANOVA with Games–Howell post hoc test), but neither whole blood nor plasma influenced its viability (*p* = 0.648 and *p* = 0.197, respectively).

## Additional information

Several methods of preparation of *S. pneumoniae* inocula for experimental infection have been outlined in the literature. The most detailed protocol that we have found uses complex media that include blood agar and Todd–Hewith broth with FCS [5]. The authors do not wash the bacteria, do not use cryoprotectants and flash freeze the inocula in liquid nitrogen. Nevertheless, this protocol may not be optimal for experimental infection models, as the presence of broth components and extracellular bacterial products might influence the biological response. Another described protocol [6] uses horse blood in the medium but it is difficult to accomplish because it requires three steps with frequent pH adjustments and several bacterial subculturing. The method that we have developed offers a wider operational window by buffering the bacterial medium, which makes the protocol much simpler, does not require the use of biological components like blood, and avoids the presence of components that may affect the biological response of interest.

Some authors wash bacteria after thawing, adding in this way an extra step that introduces variability [7]. Other researches use fresh cultures and quantify the bacteria by turbidity [8] but viability of the bacteria is not assessed and the preparation of the inocula takes much time. We propose to wash the bacteria before freezing allowing an excellent reproducibility of the stocks. The viability of the bacteria frozen at −80 °C decreases over time, although slowly [3], and we recommend to repeat the quantification of old stocks. Regarding freezing conditions it has been reported that bacteria kept at −20 °C lose viability in few weeks [3] and it is essential to store them at ≤−80 °C, an observation that we have confirmed (data not shown). The protocol that we propose is simple, inexpensive, may be finished in a working day and allows the storage for many months of highly reproducible stocks of well quantified bacteria.

Together with animal models, whole blood infection was used before isolation of highly purified blood cells was feasible [9]. Robertson and Cornwell found that mixtures of serum and leukocytes exhibited variable bactericidal activity against different *S. pneumoniae* strains, using a cumbersome technique that relied on turbidity of the bacterial culture rather than CFU [10]. We have reproduced the results of this classical work using whole blood, but have also found that plasma devoid of leukocytes show a higher bactericidal activity than blood, an observation that we are not aware to have been reported for *S. pneumoniae*. The microbicidal activity of plasma has already been described for several other bacterial species but, in sharp contrast with our results, the authors did not observe differences between serum and plasma in the antimicrobial activity [11]. We have showed that serum does not kill *S. pneumoniae*, although it retains the capacity to eliminate *E. coli*, as an example of Gram-negative microorganisms that are sensitive to serum [12]. Nevertheless, other authors have reported that *Klebsiella pneumoniae* [13] or *Helicobacter pylori* [14], both Gram-negative pathogens, are very sensitive to plasma.
